# Dietary Natural Plant Extracts Can Promote Growth and Modulate Oxidative Status of Senegalese Sole Postlarvae under Standard/Challenge Conditions

**DOI:** 10.3390/ani11051398

**Published:** 2021-05-14

**Authors:** Maria J. Xavier, Luís E. C. Conceição, Luisa M. P. Valente, Rita Colen, Andreia C. M. Rodrigues, Rui J. M. Rocha, Luísa Custódio, Carlos Carballo, Manuel Manchado, Sofia Engrola

**Affiliations:** 1 CCMAR, Centro de Ciências do Mar, Campus de Gambelas, Universidade do Algarve, 8005-139 Faro, Portugal; mmxavier@ualg.pt (M.J.X.); rcolen@ualg.pt (R.C.); lcustodio@ualg.pt (L.C.); 2 SPAROS Lda., Área Empresarial de Marim, Lote C, 8700-221 Olhão, Portugal; luisconceicao@sparos.pt; 3 CIIMAR, Centro Interdisciplinar de Investigação Marinha e Ambiental, Universidade do Porto, Terminal de Cruzeiros do Porto de Leixões, Avenida General Norton de Matos, S/N, 4450-208 Matosinhos, Portugal; lvalente@icbas.up.pt; 4 ICBAS, Instituto de Ciências Biomédicas de Abel Salazar, Universidade do Porto, Rua Jorge Viterbo Ferreira, 228, 4050-313 Porto, Portugal; 5 CESAM, Centro de Estudos do Ambiente e do Mar, Departamento de Biologia, Universidade de Aveiro, 3810-193 Aveiro, Portugal; rodrigues.a@ua.pt (A.C.M.R.); ruimirandarocha@ua.pt (R.J.M.R.); 6 IFAPA, Centro El Toruño, Junta de Andalucía, Camino Tiro Pichón, S/N, 11500 El Puerto de Santa María, Spain; carlos.carballo@juntadeandalucia.es (C.C.); manuel.manchado@juntadeandalucia.es (M.M.)

**Keywords:** plant extracts, nutrition, Senegalese sole, oxidative stress, growth performance

## Abstract

**Simple Summary:**

Oxidative stress has a direct impact on the welfare of fish, affecting growth performance and health status. Natural plant extracts present a high antioxidant capacity, due to a diversity and abundant content of polyphenols. Thus, the aim of this work was to identify if plant extracts, such as curcumin, green tea, and grape seeds, can promote oxidative status, and ultimately, enhance the growth and physiological stress response of postlarvae. Our results showed that plant extracts can improve the growth and oxidative status of the fish. Moreover, they may help fish to cope under stressful conditions. Dietary formulations with natural supplements may be a viable strategy to improve fish robustness during early life stages, and can therefore contribute to the development of aquafeeds and promote the sustainability of aquaculture production.

**Abstract:**

Plant extracts are known for their high content and diversity of polyphenols, which can improve fish oxidative status. A growth trial with Senegalese sole postlarvae (45 days after hatching) fed with one of four experimental diets—control (CTRL), and supplemented with curcumin (CC), green tea (GT), and grape seed (GS) extracts—was performed to assess if supplementation could improve growth performance and oxidative status. At the end of the growth trial, postlarvae were submitted to a thermal stress to assess their robustness. Sole growth was improved by CC and GS diets when compared to those fed the CTRL. CC and CTRL postlarvae presented the lowest oxidative damage (lipid peroxidation and protein carbonylation values). Stress-related biomarkers (heat shock protein 70 and glutathione-S-transferase) decreased in CC fish compared to those fed the CTRL diet, which might be due to a direct antioxidant capacity. In contrast, oxidative damage increased in GT and GS sole reared in standard conditions. However, after a thermal stress, GT and GS diets prevented the increase of protein carbonylation content and the decrease of antioxidant glutathione, depending on exposure time. Overall, dietary supplementation with natural extracts modulated oxidative status and stress response after a short/long-term exposure to temperature.

## 1. Introduction

Assessing fish oxidative status is crucial to improving welfare and promoting cost-effectiveness in aquaculture, as it has a direct impact on the health, growth, and flesh quality of the farmed fish.

Natural extracts from plants are an important source of bioactive compounds such as polyphenols, alkaloids, and terpenoids. These compounds are produced as secondary metabolites to protect the cells against microbial infections, exerting several biological properties, mainly antioxidant and anti-inflammatory [1,2,3,4,5]. Polyphenols are an abundant and diverse group. The two main classes are represented by phenolic acids and flavonoids [6].

Several plant extracts rich in polyphenolic compounds are considered promising feed additives in fish nutrition with high potential to enhance weight gain, feed efficiency, and/or disease resistance in aquaculture fish [7,8]. Curcumin is a lipophilic polyphenol extracted from the rhizome of turmeric (*Curcuma longa* L.)—commonly used as a spice [9]. Recently, it was reported that 1% and 1.5% of curcumin supplementation in carp (*Cyprinus carpio*) diets significantly improved growth performance, oxidative status, and skin immune response [10]. A similar effect on growth and non-specific immune response was also observed in Wuchang bream (*Megalobrama amblycephala*) fed with a diet supplemented with 0.01% curcumin [11]. In tilapia (*Oreochromis mossambicus*), the dietary administration of 0.5% and 1% curcumin modulated the expression of growth factors (insulin-like growth factors 1 and 2) in muscle and increased the activity of digestive enzymes [12]. Moreover, the inclusion of curcumin (0.01–0.04%) in diets for tilapia and rainbow trout (*Oncorhynchus mykiss*) improved oxidative status and disease resistance in response to stressful conditions [13,14,15].

Green tea (*Camellia sinensis* L.) and grape seed (*Vitis vinifera* L. *ssp sativa*) are also two plants rich in polyphenols. Green tea is one of the most popular beverages worldwide, and it contains high levels of flavanols (e.g., catechin, epigallocatechin gallate) that represent 25–35% of the leaves’ dry weight. In aquaculture production, the inclusion at 5% of green tea in juvenile grass carp (*Ctenopharyngodon idellus*) diets positively modulated myogenic regulator factors and stress-related genes [16], and at 0.01% of dietary inclusion enhanced the antioxidant and immune system in rainbow trout [17]. Grape seed extract is a by-product of the winery and grape juice industry. Polyphenols represent 5–8% of the constituent of grapes depending on the variety, and the most abundant are flavonoids, tannins, and phenolic acids [18,19]. Dietary grape seed extract at 0.1% was beneficial for growth performance and meat quality in juvenile rainbow trout [20], and in postlarvae of Senegalese sole (*Solea senegalensis*), the dietary administration of grape seeds supplemented in the mineral premix at 1.2% also showed an increase in growth performance and modulation of gene expression relate to muscle growth development [21]. All the modulatory effects observed in the growth and health of these fish species seem to be related to the antioxidant activity exhibited by polyphenol-rich extracts, which reduce the damage caused by reactive oxygen species (ROS), either by the presence of the phenol group that directly scavenge these molecules into stable radicals, or by enhancing the activity of endogenous antioxidant enzymes through an up-regulation of transcription factor *Nrf2* signalling pathway [7,22,23].

A high concentration of free radicals or other pro-oxidants that were not detoxified and removed from cells, or a deficiency in the antioxidant system, may cause an imbalance in the redox homoeostasis of the cell leading to a state of oxidative stress [24]. To counteract the high levels of ROS, all aerobic organisms possess endogenous antioxidant molecules able to neutralize them, thus preventing membrane cell damage, enzyme inactivation, and nucleic acid alterations [25]. The first line of the antioxidant defence is constituted by three enzymes: superoxide dismutase (SOD), catalase (CAT), and glutathione peroxidase (GPx) [26]. Glutathione-S-transferase (GST) acts in the second line of antioxidant defences, and is also an antioxidant enzyme family that inactivates secondary metabolites and lipid peroxidation (LPO) products into excretable molecules by catalysing the conjugation with glutathione (GSH). Other endogenous non-enzymatic antioxidant defences are also vital to maintain oxidative balance, such as vitamins (E, C, and A), ubiquitol and GSH. This previous is a peptide with an important role in the neutralization of radicals, cofactor for several detoxifying enzymes (such as GPx and GST) and converting vitamin C and E into their active forms [24,27].

Many factors can influence the antioxidant defence response in fish. Biotic factors such as age, species, and feeding behaviour, and abiotic conditions such as temperature, diet, dissolved oxygen, and toxins present in the water can modulate the antioxidant defences, and as a consequence, the oxidative status of the animals [28,29]. Therefore, the aim of this work was to evaluate if dietary plant extracts supplementation (curcumin, green tea, and grape seed) positively modulates growth performance and antioxidant status of Senegalese sole postlarvae, and to assess how the dietary treatments modify the fish’s physiological responses under a stressful event.

## 2. Materials and Methods

### 2.1. Experimental Diets

Four diets were tested in this study, including a commercial diet (WINFlat, SPAROS Lda., Portugal) that was used as the control (CTRL diet). This diet contains ingredients such as krill meal, squid meal, wheat gluten, fish meal, shrimp meal, fish hydrolysate, pea protein concentrate, fish gelatine, fish oil, lecithin, and a micronutrient premix comprising vitamins, minerals, and other additives. Moreover, three experimental diets were prepared by supplementing the CTRL diet with an extract of either curcumin (CC diet) at 46 g/kg of the micronutrient premix, green tea (GT diet) at 12 g/kg of the micronutrient premix, or grape seed (GS diet) at 12 g/kg of the micronutrient premix. The extract of curcumin (95.34% purity), green tea (≥50% polyphenols), and grape seed (≥70% polyphenols) used in the supplemented diets were provided by Denk Ingredients (Germany). These selected doses of each antioxidant extract are under a patent-pending application (PCT/IB2020/056001) and were chosen based on preliminary trials conducted in the Centre of Marine Science of Algarve (CCMAR) [21]. All diets were prepared by SPAROS Lda (Portugal). Feed samples were freeze-dried, ground, and analyzed for dry matter (DM; 105 °C for 24 h), crude protein by automatic flash combustion (Leco FP-528, Leco; N × 6.25), lipid content by petroleum ether extraction using a Soxtherm Multistat/SX PC (Gerhardt; 150 °C), and gross energy in an adiabatic bomb calorimeter (Werke C2000; IKA). Diets’ proximal composition was identical for all 4 diets, with 65.6% DM crude protein, 19.4% DM crude fat, and 22.9 MJ kg^−1^ gross energy. These diets only changed in the supplementation with natural extracts, and this supplementation did not exceed 1% of the diets.

#### 2.1.1. Preparation of Diet Extracts for Antioxidant Capacity Assessment

Methanol extracts were prepared from the four diets, CTRL, CC, GT and GS. For that purpose, the diets were freeze-dried, mixed with methanol (1:40, w/v), and maintained in an ultrasonic bath for 30 min. Then, samples were extracted overnight, with stirring, at room temperature (RT, approximately 20 °C) [30]. The extracts were then filtered (Whatman n° 4) to remove solid debris, and methanol was removed by using a rotary evaporator (60 °C; 337 mbar). The obtained dried extracts were weighed, dissolved in methanol at 50 mg/mL, and stored at −20 °C.

#### 2.1.2. Radical Scavenging Activity

Methanol extracts from diet samples were tested for radical scavenging activity against the 2,2-Diphenyl-1-picrylhydrazyl (DPPH) and 2,2′-Azino-bis-(3-Ethylbenzothiazoline-6-Sulfonic Acid) (ABTS) radicals at concentrations of 50 mg/mL, as described previously [30]. Ascorbic acid was used as a positive control at the same concentrations of the samples. Results were expressed as a percentage of inhibition, relative to a control containing methanol in place of the sample.

#### 2.1.3. Total Phenolic (TPC) and Flavonoids (TFC) Content

The TPC and TFC were determined in the methanol extracts at the concentration of 50 mg/mL, and absorbance was measured in a microplate reader (Biotek Synergy 4). The TPC was assessed by the Folin–Ciocalteu assay and TFC was estimated by the aluminum chloride colorimetric method adapted to 96-well microplates. Results were expressed, respectively, as gallic acid equivalents (GAE) and quercetin equivalents (QE) in milligrams per gram of diet (dry weight, DW). All methods were performed as previous described [30].

### 2.2. Senegalese Sole Husbandry and Experimental Set-Up

Senegalese sole postlarvae were reared for 25 days, starting at 45 days after hatching (DAH), in a recirculating aquaculture system at CCMAR (Portugal), under optimized environmental and zootechnical conditions. Sole postlarvae were kept in flat-bottom tanks (21 L), with each tank stocking 630 individuals (corresponding to a 3000 ind m^−2^). The dietary treatments (CTRL, CC, GT, and GS) were randomly assigned to replicate tanks (n = 3 tanks per treatment). Abiotic parameters were measured, and mortality was recorded daily. Dead fish were removed, and the rearing units were carefully cleaned with minimal disturbance. Dissolved oxygen in water was maintained at 96.6 ± 7.2% of saturation, temperature at 19.6 ± 0.5 °C, and salinity at 35.4 ± 0.7 g·L^−1^. A 10:14 h light:dark photoperiod was maintained, and the light intensity was 400 lx at the water surface. Inert diet was delivered semi-continuously with automatic feeders. The amount of feed distributed to each tank was based on predicted maximum growth, and daily adjustments were done based on visual inspection to avoid a large excess of uneaten food [31].

### 2.3. Thermal Stress Test

A thermal stress test was conducted at the end of the growth period (70 DAH) to assess how the dietary treatments modify the fish physiological responses. Two challenging periods were analyzed in fish response: an acute stress (at 72 DAH) and a chronic stress (at 78 DAH). The seawater temperature of the rearing system was raised from 19.6 °C until 24.0 ± 0.5 °C (~5 °C over the growth temperature) in a 24 h period. For the acute stress challenge, fish were sampled after remaining at the new temperature for 24 h (72 DAH), whilst for the chronic stress test, fish were maintained at the elevated temperature for one week (78 DAH). At the end of each thermal stress (72 and 78 DAH), fish from the different dietary treatments were sampled for biomarkers response assessment.

### 2.4. Key Performance Indicators

At the beginning (45 DAH, *n* = 60) and at the end of the growth experiment (70 DAH, *n* = 120), fish were euthanized by using an overdose of anesthetic 2-phenoxyethanol (1000 ppm; Prolabo), then individually collected for dry weight (DW, mg) and body standard length (SL, mm) determination. The postlarvae were frozen at −80 °C, photographed for measuring SL using Axio Vision L.E. 4.8.2.0 (Carl Zeiss Micro Imaging GmbH, Göttingen, Germany), and freeze-dried for DW determination (Denver Instrument, 0.001 mg precision). Survival rate (%) was calculated as the percentage of fish counted at the end of the trial relative to their initial number in each replicate. Growth, expressed as relative growth rate (RGR, %/day), was calculated, at the end of the experiment, using the following formula [32]:

(e^g−1^) × 100, with g = [(ln final weight − ln initial weight)/time] (1)

### 2.5. Preparation of Fish Sample for Physiologycal Biomarkers Response

For biomarkers analysis, 3 pools (*n* = 3 postlarve/pool) per replicate (*n* = 9 per dietary treatment) were sampled at the end of the growth trial (70 DAH) and thermal stress (72 and 78 DAH). The region from the operculum cavity until the end of the visceral cavity was selected in each fish. Samples were homogenized through sonication (Brason Sonifier 250) on ice using 1500 µL of ultra-pure water. From each sample, 2 aliquots of the supernatant were taken. One aliquot of 200 µL containing 4 µL of 4% butylated hydroxytoluene (BHT) in methanol, to prevent oxidation of lipids, was used for the determination of endogenous lipid peroxidation (LPO). The other aliquot of 500 µL was diluted (1:1) with 0.2 M K-phosphate buffer, pH 7.4, and centrifuged for 10 min at 10,000× *g* (4 °C). The post-mitochondrial supernatant (PMS) was divided into microtubes and kept at −80 °C until further analyses. All biomarkers were determined spectrophotometrically, in micro-assays set up in 96-well flat bottom plates, with the microplate reader MultiSkan Spectrum (Thermo Fisher Scientific).

#### 2.5.1. Oxidative Status Assessment

Protein concentration of PMS was determined according to the Bradford method [33], using bovine γ-globulin as a standard. Catalase (CAT) activity was determined in PMS by measuring the decomposition of the substrate 30% H_2_O_2_ at 240 nm [34]. Glutathione-S-transferase (GST) activity was determined following the conjugation of GSH with 1-chloro-2,4- dinitrobenzene (CDNB) at 340 nm (ε = 9.6 × 10^3^ M^−1^cm^−1^) [35]. Total glutathione (GSH) content was determined at 412 nm using a recycling reaction of reduced GSH with 5,5′-dithiobis-(2-nitrobenzoic acid) (DTNB) in the presence of glutathione reductase (GR) excess [36,37]. GSH content was calculated as the rate of TNB^2−^ formation with an extinction coefficient of DTNB chromophore formed (ε = 14.1 × 10^3^ M^−1^cm^−1^) [36,38]. Lipid peroxidation (LPO) was determined spectrophotometrically by measuring thiobarbituric acid-reactive substances (TBARS) [39]. Briefly, 100 μL of cold trichloroacetic acid (TCA) 100% was added to the samples, followed by 1000 μL of 2-thiobarbituric acid (TBA) 73%, and they were incubated at 100 °C for 1 h. After this period, samples were kept for up to 16 h in the dark, then were centrifuged at 10,350× *g* for 5 min at 25 °C, and 300 μL of the resulting supernatant was loaded into a microplate and absorbance was read at 535 nm. Protein carbonylation (PC) was measured by the quantification of carbonyl groups based on the reaction of 2,4-dinitrophenylhydrazine (DNPH) with carbonyl groups, according to the DNPH alkaline method [40]. The amount of carbonyl groups was quantified spectrophotometrically at 450 nm (ε = 22,308 mM^−1^cm^−1^). Total antioxidant capacity (TAC) was assessed by the decolorized radical cation ABTS by antioxidants according to their concentrations and antioxidant capacities. This change in color was measured as a change in absorbance at 660 nm, and the assay was calibrated with Trolox [41].

#### 2.5.2. Cellular Stress Response

Heat shock proteins HSP70/HSC70 content was assessed by ELISA, adapted from Rosa [42]. Samples were added to coated 96-well microplates and allowed to incubate overnight at 4 °C. Microplates were then washed (3×) with 0.05% PBS-Tween-20, and the blocking solution (1% BSA, Sigma-Aldrich, St. Louis, MO, USA) was added and left to incubate at RT for 2 h. Microplates were washed, and 5 μg mL^−1^ primary antibody (1° Anti-HSP70 mouse mAB (C92F3A-5) Millipore), detecting 72 and 73 kDa proteins corresponding to the molecular mass of inducible hsp and hsc70, was added to each well and then incubated overnight at 4 °C. The non-linked antibodies were removed by washing the microplates again, which were then incubated for 2 h at RT with 1 μg mL^−1^ of the secondary antibody, anti-mouse IgC (2° Anti-mouse IgC (fab specific) Sigma-Aldrich). After another wash, the substrate p-nitrophenyl phosphate was added and incubated for 30 min at RT. Then, the stop solution (3 mol L^−1^ NaOH) was added to each well and the absorbance was read at 405 nm, using as a standard the purified HSP70 active protein (HSP70 protein Millipore).

### 2.6. Reverse Transcription–Quantitative Real-Time PCR (qPCR) Analyses

Gene expression analysis was performed in 70, 72, and 78 DAH soles from each dietary treatment. Fish were kept at −80 °C until analysis. A region from the operculum cavity until the end of the visceral cavity was selected in each larva (*n* = 4 per dietary treatment). This process was realized in each larva without thawing. Selected genes for oxidative stress defenses and cellular stress proteins are described in detail in Table S1. Samples were homogenized using a Fast-prep FG120 instrument (Bio101) and Lysing Matrix D (Q-Bio-Gene) with 1 mL Tri Reagent (Sigma-Aldrich) for 60 s at speed setting 6. Chloroform (0.2 mL) was added to each sample before centrifuging at 11,000 × *g* for 15 min. The supernatant content was transferred to columns of the Isolate II RNA Mini Kit (Bioline) and total RNA was treated twice for 30 min with DNase I following the manufacturer’s protocols. Total RNA quality was checked by agarose gel electrophoresis and a Nanodrop ND-8000 (Thermo Scientific, Waltham, MA, USA) was used to determine its concentration. One μg of total RNA was reverse-transcribed using the iScript^TM^ cDNA Synthesis kit (Bio-Rad, Hercules, CA, USA) according to the manufacturer’s protocol.

The qPCR assays were performed in duplicate in a 10 μL volume containing cDNA generated from 10 ng of the original RNA template, 300 nM of each specific forward and reverse primer, and 10 µL of iQ™ SYBR^®^ Green Supermix (Bio-Rad). The genes analyzed were involved in the oxidative stress defenses: catalase (*cat*), glutathione peroxidase 1 (*gpx1*), glutathione peroxidase 3 (*gpx3*), superoxide dismutase [Cu-Zn] (*sod3*), and cellular stress proteins (heat shock protein 70 (*hsp70*), heat shock protein 90 alpha (*hsp90aa*), and heat shock protein 90 beta (*hsp90ab*)). Primers for Senegalese sole hsp70, hsp90aa, and hsp90ab were previously published [43,44], and new species-specific primers for qPCR were designed for remaining genes (Table S1). The qPCR amplification protocol was as follows: 7 min for denaturation and enzyme activation at 95 °C, followed by 40 cycles of 30 s at 95 °C and 1 min at 60 °C. Expression data were normalized using the geometric mean of two reference genes, ubiquitin (*ubi*) and glyceraldehyde-3-phosphate dehydrogenase 2 (*gadph2*) [45] and the relative mRNA expression calculated using the comparative Ct method [46].

### 2.7. Data Analysis

All data were tested for normality using a Kolmogorov–Smirnov (whenever *n* > 30) or Shapiro–Wilk (whenever *n* < 30) test, and for homogeneity of variance using a Levene’s test, using STATISTICA v13 (StatSoft). Data expressed as a percentage were arcsine square root transformed previously to the statistical analysis [47]. Comparisons between groups fed different diets were made using one-way ANOVA, followed by a Tukey post-hoc test for growth performance and oxidative stress biomarkers at the end of the growth trail at *p* < 0.05 level of significance. To assess the response of oxidative stress biomarkers to thermal stress exposure by each treatment group, a two-way ANOVA was made. The analysis of the delta variation between pre- and post-stress indicators was performed by a one-way ANOVA, both followed by a Tukey post-hoc test. Significance levels were set at *p* < 0.05.

## 3. Results

### 3.1. Dietary Antioxidant Properties

Radical scavenging activity (RSA) in the experimental diets was low to moderate (Table 1). Regarding the ABTS assay, the GS diet presented the highest activity (28.6% for ABTS), while in the DPPH method, the utmost activity (50.2%) was recorded for the CC diet. The TPC ranged from 1.3 to 6.4 mg GAE/g diet, and the three supplemented diets had higher levels than CTRL (*p* < 0.001). The content of flavonoids varied from 0.7 to 4.4 mg QE/g diet. The GS diet presented the highest amounts of flavonoids, similar to the CC diet (*p* < 0.001). On average, the total phenols and flavonoids content in the supplemented diets was 4-fold higher than in the CTRL diet.

### 3.2. Key Performance Indicators

**Figure 1:**
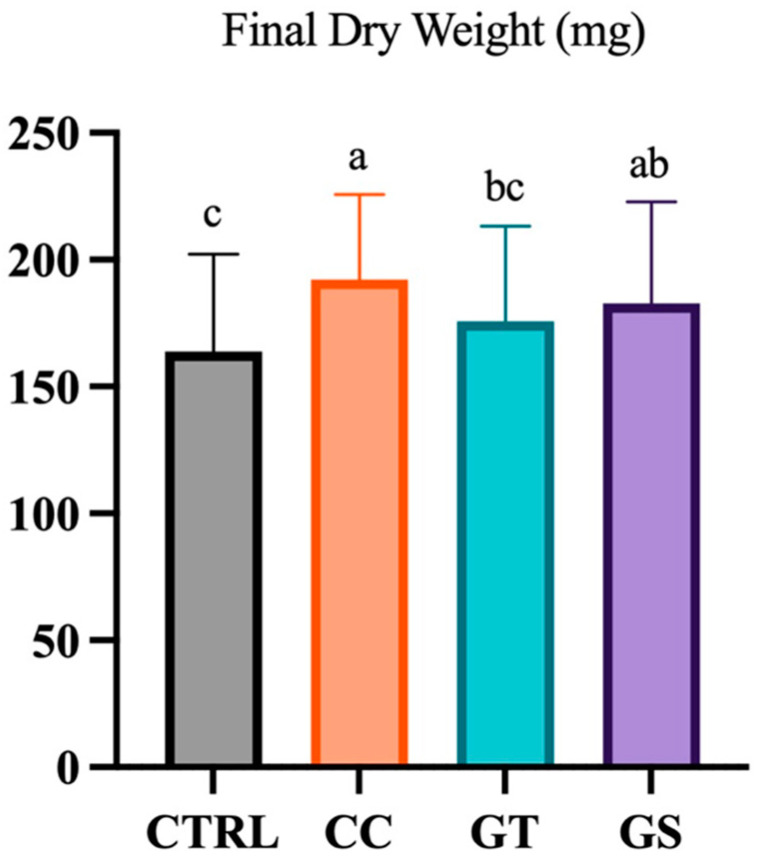
Dry weight (DW, mg) of Senegalese sole fed different diets (CTRL, CC, GT, and GS) at the end of the growth trial (70 DAH). Values are present as means ± SD. Different superscript letters indicate significant differences (*p* < 0.05, 1-way ANOVA) between the dietary treatments.

At the beginning of the experiment, postlarvae DW and ST was 12.3 ± 5.7 mg and 17.7 ± 3.1 mm, respectively. Postlarvae fed with CC and GS diets were heavier than those fed with the CTRL diet at the end of the feeding trial (*p* < 0.001) (Figure 1). Moreover, all sole fed with supplemented diets (CC, GT, and GS) showed a higher length than CTRL group (*p* < 0.001). Relative growth rate (RGR) was around 11.3 ± 0.5% day^−1^ from 25 to 70 DAH. The average survival rate was 98.3 ± 0.6% and did not differ between dietary treatments (*p* = 0.460).

### 3.3. Oxidative Status and Cellular Stress Indicators

**Figure 2:**
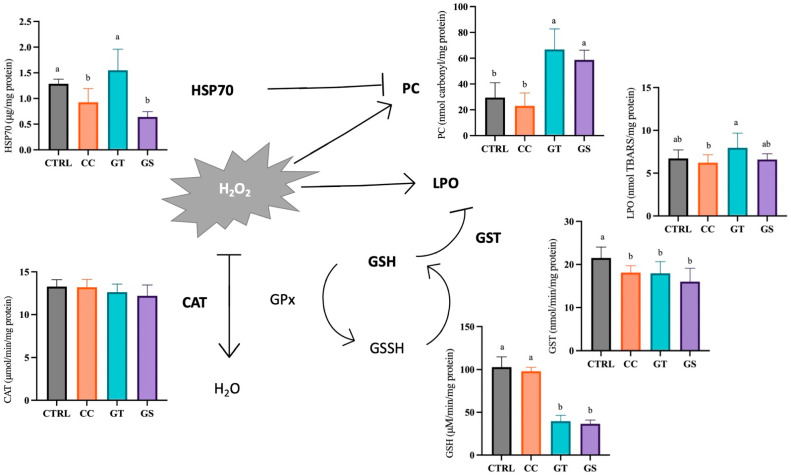
Schematic (simplistic) representation of the response and pathways of several oxidative stress-related biomarkers of 70 DAH Senegalese sole fed with different diets at the end of the growth trial (standard condition). Values are present as means ± SD. Different superscript letters indicate significant differences (*p* < 0.05, 1-way ANOVA) between the dietary treatments.

At the end of the growth trial, the CAT activity and TAC showed similar values between dietary treatments, around 12.8 ± 1.1 μmol/min/mg protein and 1.30 ± 0.3 μM trolox equivalents/mg protein, respectively (*p* > 0.05). Postlarvae fed with all the supplemented diets showed a lower GST activity than CTRL (*p* = 0.001). The content of GSH was significantly higher in sole from dietary CC and CTRL treatments (*p* < 0.001). When HSP70 proteins were analyzed, sole fed with CC and GS had lower levels than CTRL and GT (*p* < 0.001). The oxidative damage measured as LPO was not significantly different between supplemented diets and the CTRL, and only a significant reduction in CC with respect to the GT diet was found (*p* = 0.024). The postlarvae from CC and CTRL diets had the lowest values of protein oxidative damage (PC) when compared with fish from GT and GS diets (*p* < 0.001) (Figure 2).

Expression of most of the genes related to oxidative stress was not modified by dietary treatments, except for *cat* and *gpx1* genes (*p* < 0.05) (Table 2). The postlarvae fed with GT had lower *cat* mRNA levels than GS. An up-regulation of *gpx1* transcription was observed in fish fed with CC and GT compared to the other dietary treatments.

#### 3.3.1. Thermal Stress—Acute Exposure

**Figure 3:**
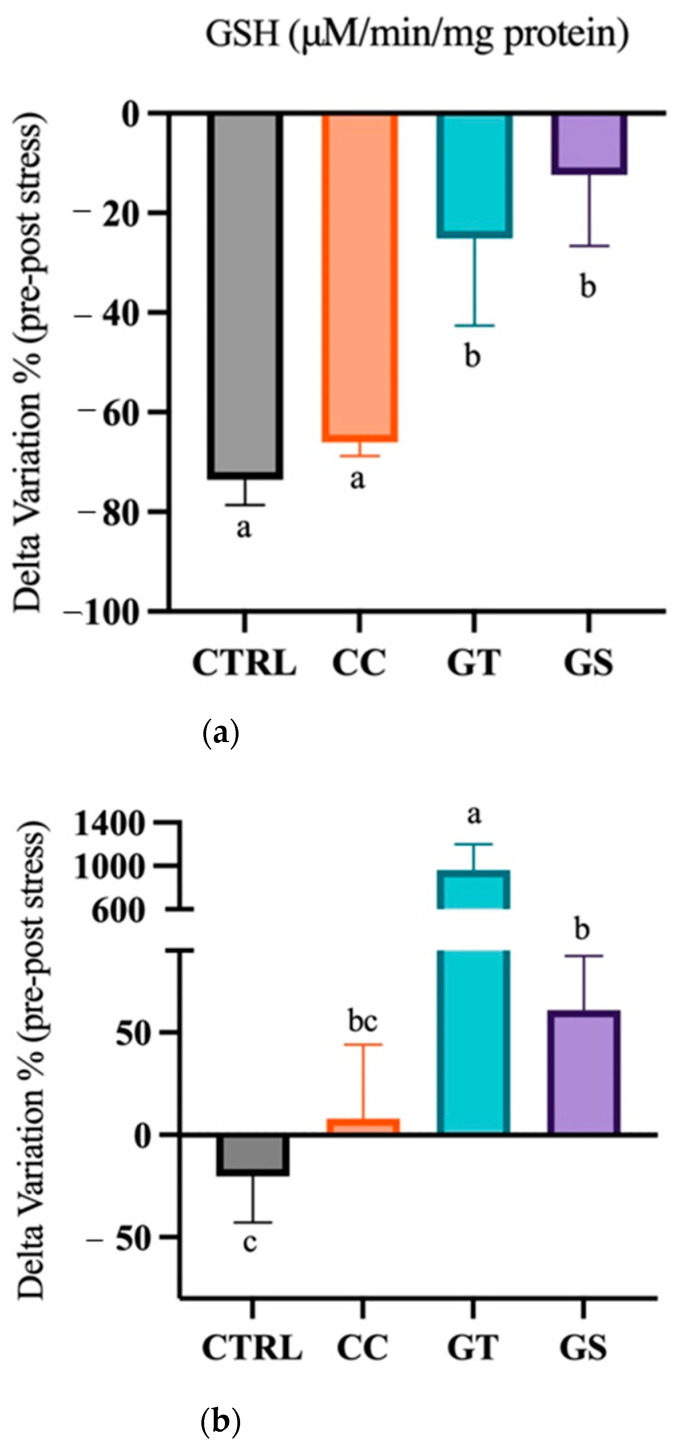

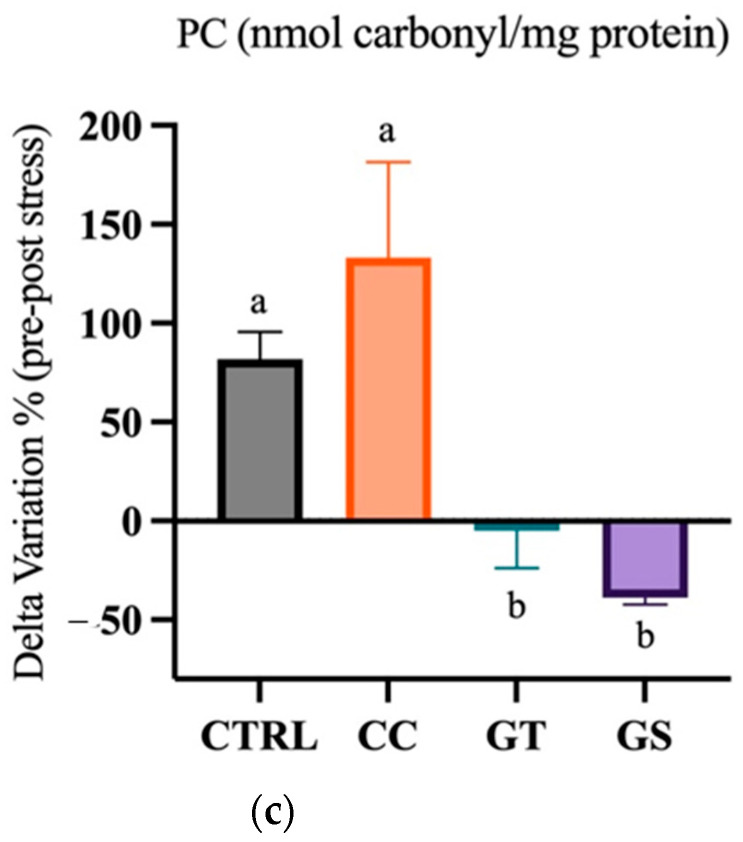
Delta variation (%) of the response of oxidative and cellular stress-related biomarkers (**a**) glutathione-s-transferase (GSH), (**b**) heat shock protein 70 (HSP70), and (**c**) protein carbonyl (PC) from standard to acute stress conditions of Senegalese sole fed with different diets (CTRL, CC, GT, and GS). Values are presented as means ± SD, *n* = 3. Different superscript letters indicate significant differences (*p* < 0.05, 1-way ANOVA) between the dietary treatments.

A significant decrease in the activity of CAT was observed in sole after acute stress (*p* = 0.024). The postlarval activity of GST did not significantly decrease in CC and GS treatments, unlike CTRL and GT postlarvae (*p* = 0.003). Moreover, the high levels of GSH present in sole from the CTRL and CC treatments after stress were significantly reduced after 24 h of thermal stress (*p* < 0.001) (Table S2). All dietary treatments revealed a reduction in GSH content, but the lowest decrease was observed in the GS and GT treatments (*p* < 0.001) (Figure 3a). The levels of HSP70 were similar after the acute stress in sole fed with the CTRL, CC, and GS diets; however, in GT, a 10-fold increase of HSP70 levels was observed after acute stress (*p* < 0.001) (Table S2). The delta variation between standard conditions/acute stress of the HSP70 levels showed a slight increase in the content in GS postlarvae compared to CTRL group (*p* < 0.001) (Figure 3b). TAC increased in postlarvae after acute stress (*p* < 0.001), and in sole fed with CC, a significantly higher TAC was observed than in postlarvae fed the GS diet (*p* = 0.026). The PC content was higher in sole fed with CTRL and CC after the acute stress (*p* < 0.001) (Table S2). Moreover, the delta variation between pre/post-stress showed that the fish fed with GT and GS were able to decrease PC content (*p* < 0.001) (Figure 3c). The postlarval LPO content were not affected by the acute increase of temperature (*p* = 0.054) (Table S2).

Postlarvae exposed to acute thermal stress showed reduced *gpx3* and *gsr* mRNA levels and increased *hsp90b* and *hsp70* transcript amounts independently of the dietary treatment (*p* > 0.05). The expression levels of *cat* decreased after acute stress; however, the fish fed with GS up-regulated the expression of *cat* compared to CTRL and GT (*p* < 0.013) (Table S2). The expression of *sod3* and *gpx1* were not modified by the thermal stress and dietary treatments (*p* > 0.05). A downregulation of *hsp90aa* mRNA levels was observed in both CC and GT soles after the acute stress, as opposed to CTRL and GS fish, in which up-regulation of the expression of this gene was observed (Table S2).

#### 3.3.2. Thermal Stress—Chronic Exposure

**Figure 4:**
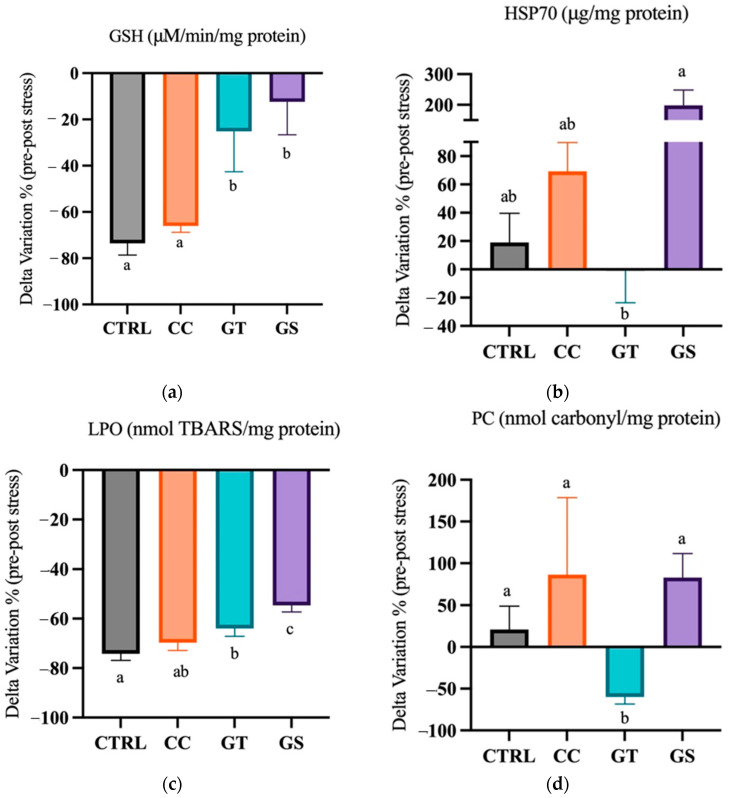
Delta variation (%) of the response of oxidative and cellular stress-related biomarkers (**a**) glutathione-s-transferase (GSH), (**b**) heat shock protein 70 (HSP70), (**c**) lipid peroxidation (LPO), and (**d**) protein carbonyl (PC) from standard to chronic stress conditions of Senegalese sole fed with different diets (CTRL, CC, GT, and GS). Values are presented as means ± SD, *n* = 3. Different superscript letters indicate significant differences (*p* < 0.05, 1-way ANOVA) between the dietary treatments.

After chronic stress, postlarvae GSH content was the highest in GS treatment compared to CTRL and CC groups (*p* < 0.001) (Table S3). The delta variation of the levels of this tripeptide, under standard rearing and chronic stress conditions, showed a decrease in all treatments; however, in GT and GS sole, this decline was smaller than in the remaining diets (*p* < 0.001) (Figure 4a). The content of HSP70, after chronic stress, was similar between all dietary treatments (Table S3). However, regarding the delta variation, a significant difference was visible between the highest values of HSP70 content in GS fish compared to GT treatment (*p* = 0.012) (Figure 4b). The PC content decreased in fish fed with GT after the chronic exposure; on the contrary, fish fed with GS presented the highest amounts (*p* < 0.001) (Table S3). The delta variation showed that CC, GS, and CTRL sole had improved PC content when compared to GT fish (*p* = 0.001) (Figure 4c). The fish activity of CAT and TAC was higher after a chronic thermal stress, and in opposition, the activity of GST and LPO content decreases after chronic exposure (*p* < 0.05) (Table S3). The delta variation in the levels of LPO between pre- and post-stress showed that reduction was higher in CTRL fish compared to GT and GS treatments (*p* < 0.001) (Figure 4d).

The expression of *gpx3*, *gsr*, *hsp90b*, and *hsp70* was only affected by the long exposure to higher temperature (*p* < 0.05), regardless of the dietary treatments (Table S3). After 7 days, all postlarvae up-regulated the expression of *hsp70* and *gpx3* and down-regulated gsr. Moreover, the cat mRNA levels increased after thermal chronic stress (*p* < 0.001) and the fish fed with GS presented a significantly higher expression than those fed with GT (*p* < 0.05). The expression of *sod1* was not affected by the dietary treatments nor thermal exposure (*p* > 0.05). The long exposure to high temperature up-regulated *gpx1* mRNA levels only in CTRL and GT postlarvae. After chronic temperature exposure, the transcription of hsp90aa in CC sole significantly decreased to values similar of those observed in the remaining treatments (Table S3).

## 4. Discussion

In the last decade, there has been a growing interest in dietary inclusion of natural extracts with high biological activity as feed additives to enhance growth performance and welfare of farmed fish species. In this work, three experimental diets supplemented with antioxidants (extracts from turmeric, green tea, and grape seed) were tested in Senegalese sole postlarvae. The present results show that diet supplementation with curcumin (CC diet) and grape seed (GS diet) extracts promote larger sole postlarvae. In fact, previous data showed that these supplemented diets improve sole growth performance through a modulation in the expression of genes related to muscle growth and development [21].

Growth and development are well-orchestrated processes that depend on the balance of cellular proliferation, differentiation, and apoptosis. The cell fate depends on a variety of molecular pathways, gene expression, and protein function, which are sensitive to the cellular reduction potential. Measuring the levels of antioxidant defenses and oxidative damage is highly relevant to assess fitness, because high oxidative stress levels may compromise survival and growth [48,49]. In order to understand the possible pathways by which different natural antioxidants modulate the antioxidant status of sole, several oxidative stress biomarkers were analyzed in the present work. The results from our study suggest that CC diet supplementation improved oxidative status in Senegalese sole postlarvae. Contradictory results were observed with the GT and GS diets that were shown to increase sole protein oxidative damage content and decrease the levels of essential non-enzymatic antioxidant GSH at the end of the growth trial. Both fish fed with CC and CTRL diet showed the lower levels of lipid and protein oxidation. However, in order to maintain these similar levels of LPO and PC, the fish from CTRL needed to increase endogenous antioxidant defense GST and HSP70, which are linked to the biotransformation process of metabolites and to the protection of the cell during stress, respectively.

Therefore, it seems that postlarvae of CTRL required a higher GST activity to detoxicate LPO products and a higher content of protein chaperone HSP70 to prevents the accumulation of oxidized proteins and promote their degradation [50]. Increasing antioxidant defenses is an energy-consuming process and may deflect energy or nutrients that might be needed for other physiological functions. Therefore, curcumin extract supplementation in diets may spare antioxidant defenses by directly scavenging the ROS. Consequently, CC postlarvae were able to allocate energy in other functions like growth. Similarly, in tilapia [51] and common carp [10], an improvement in fish final weight when feeding a lower dose of curcumin supplementation was reported. However, no differences were registered in the content of lipid peroxidation and activity of endogenous antioxidants defenses when compared with the control treatment. Moreover, in a test performed with climbing perch (*Anabas testudineus*) fed with two doses of curcumin for 8 weeks, unaltered levels of endogenous enzymes (GR, CAT, and GPx) and oxidative damage LPO were observed in supplemented fish compared to the control [52], also suggesting that curcumin has a direct free radical scavenging activity. In juveniles of tilapia, the growth improvement observed in fish fed the higher dose of curcumin was accomplished by a decrease of LPO, but with no differences in the activity of CAT and GSH content [15]. Nevertheless, other studies reported that supplementation of curcumin enhanced growth of tilapia and trout juveniles, but with a decrease in the LPO content and improvement in the activity of antioxidant defenses [14,53]. Given the multitude of pathways that curcumin can act on, it is challenging to identify the key pattern of action of this antioxidant. In fact, the impact of curcumin in the redox status of fish is highly dependent on the duration of the experimental trial [52], doses [10,15], and species [54].

It appears that the levels of supplementation of both green tea (GT diet) and grape seed (GS diet) in the present study might have a pro-oxidant effect in sole postlarvae. Although there are just a few studies regarding the pro-oxidant effects of antioxidants, a variety of plant extracts have been shown to have both pro-oxidative and antioxidative properties, depending on their main bioactive molecules characteristics (e.g., metal-reducing potential, chelating behavior), concentration, and the microenvironment (e.g., pH and presence of metal ions and redox status) [55,56]. As far we know, there are no previous studies regarding the pro-oxidant effect of either green tea or grape seed extracts in the oxidative status of health animal models. However, juveniles of rainbow trout fed with grape seed oil supplementation [57], at higher concentrations, also showed an improvement in growth and a decreased activity of some endogenous antioxidant defenses (SOD, CAT, and GST), which might corroborate the present results. Moreover, the inclusion of Epigallocatechin-3-gallate (catechin present in green tea) and green tea in diets for rainbow trout juveniles [58] and grass carp [16], respectively, did not affect the oxidative status parameters analyzed. On the other hand, rainbow trout juveniles fed green tea presented a higher SOD activity and lower LPO content compared to the control group [17]. A similar pattern was observed when rainbow trout were fed dietary grape seed extracts, showing a reduction of LPO and upregulation of antioxidant defenses [59]. Hence, the inclusion of green tea and grape seed extracts in fish diets can improve the oxidative status and consequently improve the endogenous antioxidant defenses in fish species. Therefore, the dose of the antioxidant supplementation and the duration of the administration period still need tuning. Moreover, it is also important to highlight that postlarvae have higher growth rates and feed intake than juveniles and adults, which might have contributed to increased concentration of antioxidants per body weight unit, possibly explaining the pro-oxidant effect observed in this study. Nevertheless, our results also corroborate previous concepts, including that polyphenols can modulate oxidative status biomarkers by different mechanisms of action [6].

High water temperatures increase oxygen consumption and mitochondrial respiratory rates, leading to an increase of proton leakage rates that could lead to oxygen incomplete reduction and ROS formation [60]. Our data suggest that sole fed with CC and CTRL diets were unable to maintain the lower PC and the highest GSH content after an acute increase of temperature. Moreover, fish fed with CTRL could not maintain the activity levels of GST after 24 h of thermal stress. On the other hand, sole fed with GS were able to maintain the activity of GST and GSH content after the acute stress, and were even able to decrease the PC levels. It seems that the pro-oxidant effect under standard rearing condition has reversed to antioxidant under acute challenging conditions. In fact, dietary pro- or antioxidant can have different impacts on the oxidative status of fish depending on normal/stressful rearing condition [61]. This may suggest that the supplementation dose needs to be tuned between promoting growth in standard rearing conditions and to help fish coping with environmental changes. In other fish species, a more evident effect of curcumin supplementation in response to a stressful situation was observed. For example, both rainbow trout [13] and tilapia [51] fed diets supplemented with curcumin showed a decrease in LPO content. Moreover, the expression of *Nrf2* and antioxidant defenses activity (SOD and GPx) were also promoted in these studies. Similarly, previous reports corroborate our study, and evidence the protective effect of grape (or grape related) extracts on induced oxidative stress. In the case of abalone fed a grape seed extract supplementation diet under a thermal stress (higher water temperature), the expression of *cat* was up-regulated compared to the control treatment. Juveniles of grass carp infected with *Pseudmonas aeruginosa* and fed grape pomace flour supplementation had an increased CAT activity in serum and splenic tissues [62]. Moreover, tilapia fed diets with different doses of resveratrol were able to counteract some negative effects of intraperitoneal injection of H_2_O_2_ in the oxidative status of the fish, by decreasing the levels of LPO and enhancing CAT activity and TAC [63].

Depending on the severity and duration of thermal stress, the antioxidant system and associated enzymes act differently [64]. After one week of thermal stress (chronical exposure), the fish might be adapted to the new conditions and restore the homeostatic values or reach an allostatic equilibrium. Overall, LPO content of the fish decreased independently of the dietary treatment, and the values of PC of the postlarvae from GS rose again. On the other hand, the values of protein oxidation in the GT sole were not affected by the chronic thermal exposure, which may indicate that a high content of HSP70, as a response to an acute stress, might have a long-term effect in the protection of proteins from oxidative stress. Similar adjustments of oxidative status and adaptive responses of antioxidant defenses to stressful events were also reported in other fish species fed with green tea extracts. The antioxidant role of the green tea was reported to remedy the toxic effects of 4-nonylphenol in African catfish, *Clarias gariepinus* [65], and the toxic effect of oxidised fish oil in sturgeon hybrid of Sterlet (*Huso huso* ♂ × *Acipenser ruthenus* ♀) [66]. In both studies, fish were able to decrease LPO content and increase the levels of some antioxidant biomarkers.

**Figure 5:**
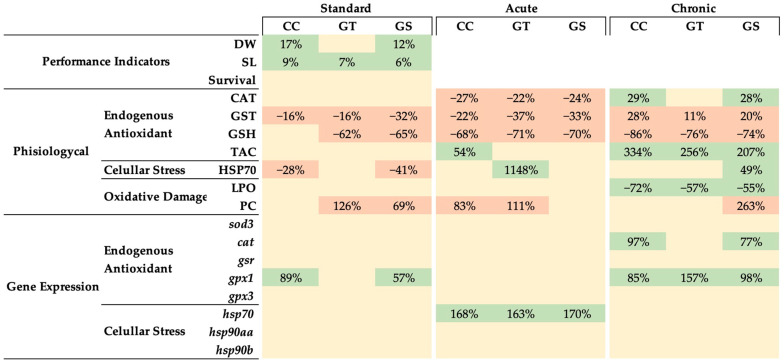
Integrative analysis of performance indicators, physiological and gene expression biomarkers of oxidative status in Senegalese sole postlarvae fed antioxidant-supplemented diets (CC, GT, and GS) during the experimental period (standard, acute, and chronic). All the data were normalized to the CTRL fish responses at standard condition (([test diet value]/[CTRL diet value] − 1) × 100). Percentages given correspond to inferior (orange color) and superior (green color) values of the different dietary treatments in relation to CTRL fish at standard condition (*p* < 0.05, 1-way ANOVA).

The dietary supplementation of natural antioxidants was an effective way of modulating the oxidative status, antioxidant response, and growth of Senegalese sole postlarvae, under different rearing conditions (Figure 5). These antioxidants are known to interfere in numerous metabolic routes, by acting as direct antioxidants on chelating metals, reducing via electron transfers or hydrogen atoms transference, and indirectly, by upregulating the expression of endogenous antioxidants. At the end of the growth trial, an increase of 12–17% in sole growth was observed in CC- and GS-fed fish compared to CTRL sole, although the mechanism by which these supplements act seem different and still requires further investigation. It seems that curcumin extract improved the oxidative status of the sole, showing low levels of protein and lipid degradation and a reduction of GST activity and HSP70 content, allowing fish to invest more energy into growth rather than on endogenous oxidative defenses. On the other hand, the positive effect of GS in sole growth might be reverted in the long term, as both dietary GS and GT seems to act as pro-oxidants by increasing the protein oxidation and decreasing some antioxidant defenses. In response to a thermal stress, both GT and GS diets increased the fish’s capacity to cope with the new stressful event. However, these antioxidants appear to act in distinct time manners and to exert different responses: grape seed extract seems to have an immediate action and result in a short-term improvement on fish oxidative status, while green tea extract has a long-term effect on the antioxidant capacity.

## 5. Conclusions

Overall, curcumin extract seems a good candidate for long-term supplementation of young fish diets, as it improves the welfare and growth of Senegalese sole. The use of green tea and grape seed extracts in diets for young fish still requires further evaluation to identify the most adequate inclusion level, although the short-term use of the tested doses seems a feasible solution before highly stressful short periods (e.g., transportation, handling, and temperature rises). These results suggest that dietary natural plant extracts can improve young fish robustness and even promote growth when supplemented at the optimal doses.

## 6. Patents

The three experimental diets (CC, GT, and GS) are included in the patent-pending application PCT/IB2020/056001.

## Figures and Tables

**Table 1:** Antioxidant properties (expressed as % of activity) and total phenolics (TPC; expressed as mg GAE/g diet) and flavonoids contents (TFC; mg QE/g diet) of methanol extracts from the experimental diets (CTRL, CC, GT, and GS).

Assay/Parameter	Experimental Diets				1-Way Anova
	CTRL	CC	GT	GS	*p* Value
ABTS (% of activity)	19.4 ± 2.4 ^b^	18.5 ± 0.9 ^b^	17.3 ± 1.4 ^b^	28.6 ± 1.3 ^a^	<0.001
DPPH (% of activity)	8.9 ± 3.2 ^c^	50.2 ± 9.9 ^a^	34.1 ± 2.0 ^b^	39.9 ± 4.1 ^b^	<0.001
TPC (mg GAE/g diet)	1.3 ± 0.6 ^b^	6.4 ± 1.9 ^a^	5.0 ± 2.1 ^a^	5.4 ± 0.9 ^a^	<0.001
TFC (mg QE/g diet)	0.7 ± 0.3 ^c^	2.9 ± 0.7 ^ab^	1.5 ± 0.2 ^bc^	4.4 ± 3.7 ^a^	<0.001

Values were expressed as mean ± SD. Different superscript letters indicate significant differences between the dietary treatments.

**Table 2:** Gene expression of oxidative and cellular stress-related biomarkers in 70 DAH Senegalese sole fed with different diets (CTRL, CC, GT, and GS) at the end of the growth trial (standard condition).

Gene Expression	Standard Condition				1-Way Anova
	CTRL	CC	GT	GS	*p* Value
*sod3*	1.0 ± 0.1	0.9 ± 0.1	0.8 ± 0.1	1.0 ± 0.1	0.446
*cat*	1.0 ± 0.1 ^ab^	1.1 ± 0.1 ^ab^	0.9 ± 0.1 ^b^	1.4 ± 0.1 ^a^	0.046
*gsr*	1.0 ± 0.1	1.3 ± 0.2	1.1 ± 0.0	1.4 ± 0.2	0.266
*gpx1*	1.0 ± 0.1 ^b^	1.9 ± 0.5 ^a^	1.1 ± 0.1 ^b^	1.6 ± 0.2 ^a^	0.047
*gpx3*	1.0 ± 0.1	0.9 ± 0.1	0.9 ± 0.1	1.1 ± 0.2	0.431
*hsp70*	1.0 ± 0.1	1.2 ± 0.2	1.3 ± 0.1	1.6 ± 0.3	0.309
*hsp90aa*	1.0 ± 0.1	3.3 ± 0.6	1.5 ± 0.5	1.8 ± 0.4	0.051
*hsp90b*	1.0 ± 0.1	1.0 ± 0.1	0.8 ± 0.0	1.0 ± 0.1	0.453

Values are presented means ± SD. Different superscript letters indicate significant differences between the dietary treatments.

## Data Availability

The dataset supporting this study are present within the article and Supplementary Materials.

## Supplementary Materials

The following are available online at https://www.mdpi.com/article/10.3390/ani11051398/s1, Table S1: Primes used in qPCR, Table S2: The response of several oxidative stress-related biomarkers of Senegalese sole fed with different diets at the end of the growth trial (standard) and after thermal acute stress (acute), Table S3: The response of several oxidative stress-related biomarkers of Senegalese sole fed with different diets at the end of the growth trial (standard) and after thermal chronic stress (chronic).
