# Supplementary material for: Dietary Natural Plant Extracts Can Promote Growth and Modulate Oxidative Status of Senegalese Sole Postlarvae under Standard/Challenge Conditions
Source: Animals (Basel). 2021 May 14;11(5):1398. doi: 10.3390/ani11051398 (PMC8156806; doi:10.3390/ani11051398)
Supplement: Supplementary file 1 [file animals-11-01398-s001.zip › animals-1218214-supplementary.pdf]

animals

Article

Dietary Natural Plant Extracts Can Promote Growth and Modulate Oxidative Status of Senegalese Sole Postlarvae under Standard/Challenge Conditions

Maria J. Xavier [1,2,3,4], Luís E.C. Conceição [2], Luisa M.P. Valente [3,4], Rita Colen [1], Andreia C.M. Rodrigues [5], Rui J.M. Rocha [5], Luísa Custódio [1], Carlos Carballo [6], Manuel Manchado [6] and Sofia Engrola [1,*]

[1] CCMAR, Centro de Ciências do Mar, Campus de Gambelas, Universidade do Algarve 8005-139 Faro, Portugal; mmxavier@ualg.pt (M.J.X.); rcolen@ualg.pt (R.C.); lcustodio@ualg.pt (L.C.)
[2] SPAROS Lda., Área Empresarial de Marim, Lote C, 8700-221 Olhão, Portugal; luisconceicao@sparos.pt
[3] CIIMAR, Centro Interdisciplinar de Investigação Marinha e Ambiental, Universidade do Porto, Terminal de Cruzeiros do Porto de Leixões, Avenida General Norton de Matos, S/N, 4450-208 Matosinhos, Portugal; lvalente@icbas.up.pt
[4] ICBAS, Instituto de Ciências Biomédicas de Abel Salazar, Universidade do Porto, Rua Jorge Viterbo Ferreira, 228, 4050-313 Porto, Portugal
[5] CESAM, Centro de Estudos do Ambiente e do Mar, Departamento de Biologia, Universidade de Aveiro, 3810-193 Aveiro, Portugal; rodrigues.a@ua.pt (A.C.M.R.); ruimirandarocha@ua.pt (R.J.M.R.)
[6] IFAPA, Centro El Toruño, Junta de Andalucía, Camino Tiro Pichón s/n, 11500 El Puerto de Santa María, Spain; carlos.carballo@juntadeandalucia.es (C.C.); manuel.manchado@juntadeandalucia.es (M.M.)
* Correspondence: sengrola@ualg.pt; Tel.: +351-289-800-900

Citation: Xavier, M.J.; Conceição,L.E.C.; Valente, L.M.P.; Colen, R.; Andreia C.M.; Rodrigues, A.C.M.; Rocha,R.J.M.; Custódio, L.; Carballo, C.; Manchado, M.; Engrola, S. Dietary Natural Plant Extracts can Promote Growth and Modulate Oxidative Status of Senegalese Sole Postlarvae under Standard/Challenge Conditions. *Animals* **2021**, *11*, 1398. https://doi.org/10.3390/ani11051398

Academic Editor: Alessia Giannetto

Received: 26 April 2021
Accepted: 11 May 2021
Published: date

Publisher's Note: MDPI stays neutral with regard to jurisdictional claims in published maps and institutional affiliations.

Copyright: © 2021 by the authors. Submitted for possible open access publication under the terms and conditions of the Creative Commons Attribution (CC BY) license (https://creativecommons.org/licenses/by/4.0/).

Supplementary Materials

animals MDPI

Table S1. Primers used in qPCR.

Gene	Fwd sequence (5′ → 3′)	Rev sequence (5′ → 3′)	Acession nr (GenBank)	Size (bp)
sod3	AGTCGGAAGCAACACTGTTT-GAAGGGATGT	GCCAGCATCTCCACCCAG-TCCTAGGTCA	unigene29222	98
cat	CCAA-GCCCCGACAAAATGCTTCAGGGT	CCACGCGGGTTCTGAATGGG-CAGTTGAC	unigene326680	118
gsr	AGATGCTTACGTT-GGCCGCCTGAACCAC	CCTCCAC-TGTCGGCTCAGGGTCATCTGT	unigene3049	116
gpx1	TGTGAACGGAG-CAGATGCACACCCCTT	AACTTTGGATCGGTCATGA-GAGCCATGGTA	unigene65687	99
gpx3	GTTGTGGACCTTT-GTGCCGCCGCTG	GCTGCACTGCCGAGTCAAC-GATGCCT	unigene29032	88
hsp70	GCTATAC-CAGGGAGGGATGGAAGGAGGG	CGACCTCCTCAATATTT-GGGCCAGCA	AB513855	119
hsp90aa	GACCAAGCCTATCTGGACCCG-CAAC	TTGACAGCCAGGTGGTCCTCCCAGT	Manchado et al., 2009	105
hsp90ab	TCAGTTTGGTGTGGGTTTC-TACTCGGCTTA	GCCAAGGGGCTCACCTGTGTCG	Manchado et al., 2009	148

Gene name, sequences, accession numbers at GenBank or SoleaDB and amplicon sizes are indicated.

Table S2. The response of several oxidative stress-related biomarkers of Senegalese sole fed with different diets (CTRL, CC, GT and GS) at the end of the growth trial (standard) and after thermal acute stress (acute).

	**CTRL**		**CC**		**GT**		**GS**		**2-way Anova**		
	**Standard**	**Acute**	**Standard**	**Acute**	**Standard**	**Acute**	**Standard**	**Acute**	**Stress**	**Diet**	**Diet*Stress**
Physiological indicators											
CAT (μmol/min/mg protein)	13.3 ± 0.8^{a}	9.2 ± 1.3^{b}	13.2 ± 0.9^{a}	9.7 ± 0.8^{b}	12.6 ± 1.0^{a}	10.3 ± 0.6^{b}	12.2 ± 1.3^{a}	10.1 ± 1.4^{b}	<0.001	0.729	0.024
GST (nmol/min/mg protein)	21.5 ± 2.5^{a}	15.6 ± 1.1^{bcd}	18.1 ± 1.6^{b}	16.7 ± 1.1^{bc}	17.9 ± 2.7^{bc}	13.6 ± 1.4^{d}	16.0 ± 3.1^{bcd}	14.4 ± 0.5^{cd}	<0.001	<0.001	0.003
GSH (μM/min/mg protein)	102.9 ± 11.9^{a}	26.3 ± 3.6^{c}	97.9 ± 4.7^{a}	33.2 ± 4.0^{bc}	39.6 ± 6.9^{b}	29.8 ± 9.3^{bc}	36.6 ± 4.4^{bc}	31.2 ± 5.0^{bc}	<0.001	<0.001	<0.001
TAC (μM Trolox equivalents/mg protein)	1.3 ± 0.2^{pxy}	1.8 ± 0.6q^{xy}	1.3 ± 0.4^{px}	2.0 ± 0.6^{qx}	1.5 ± 0.4^{xpy}	1.7 ± 0.2q^{xy}	1.1 ± 0.1^{py}	1.3 ± 0.4^{qy}	<0.001	0.026	0.273
HSP70 (μg/mg protein)	1.3 ± 0.1^{b}	1.0 ± 0.4^{b}	0.9 ± 0.3^{b}	0.9 ± 0.1^{b}	1.6 ± 0.4^{b}	16.1 ± 2.0^{a}	0.6 ± 0.1^{b}	1.0 ± 0.2^{b}	<0.001	<0.001	<0.001
LPO (nmol TBARS/mg protein)	6.7 ± 1.0^{xy}	6.6 ± 0.8^{xy}	6.2 ± 0.9^{y}	5.3 ± 0.9^{y}	8.0 ± 1.7^{x}	6.9 ± 1.5^{x}	6.6 ± 0.7^{xy}	6.4 ± 1.1^{xy}	0.054	0.001	0.512
PC (nmol carbonyl/mg protein)	29.5 ± 11.4^{c}	55.8 ± 14.1^{ab}	23.0 ± 10.1^{c}	53.8 ± 20.7^{ab}	66.7 ± 16.0^{a}	62.2 ± 21.9^{a}	58.7 ± 7.5^{a}	35.8 ± 10.6^{bc}	0.041	<0.001	<0.001
Gene expression											
sod3	1.0 ± 0.1	0.8 ± 0.1	0.9 ± 0.1	0.9 ± 0.1	0.8 ± 0.1	0.7 ± 0.1	1.0 ± 0.1	0.8 ± 0.2	0.116	0.741	0.774
cat	1.0 ± 0.1^{py}	0.7 ± 0.2^{qy}	1.1 ± 0.1^{pxy}	0.9 ± 0.1^{qxy}	0.9 ± 0.1^{py}	0.8 ± 0.1^{qy}	1.4 ± 0.1^{px}	1.1 ± 0.1^{qx}	0.006	0.013	0.794
gsr	1.0 ± 0.1^{p}	0.8 ± 0.0^{q}	1.3 ± 0.2^{p}	1.0 ± 0.2^{q}	1.1 ± 0.0^{p}	0.9 ± 0.1^{q}	1.4 ± 0.2^{p}	1.1 ± 0.1^{q}	0.011	0.145	0.908
gpx1	1.0±0.1	1.7 ± 0.2	1.9 ± 0.5	1.5 ± 0.5	1.1 ± 0.1	0.9 ± 0.1	1.6 ± 0.2	1.5 ± 0.3	0.884	0.050	0.076
gpx3	1.0±0.1^{p}	1.0 ± 0.2^{q}	0.9 ± 0.1^{p}	0.7 ± 0.2^{q}	0.9 ± 0.1^{p}	0.6 ± 0.1^{q}	1.1 ± 0.2^{p}	0.7 ± 0.1^{q}	0.009	0.138	0.481
hsp70	1.0 ± 0.1^{p}	2.5 ± 0.7^{q}	1.2 ± 0.2^{p}	2.7 ± 0.0^{q}	1.3 ± 0.1^{p}	2.6 ± 0.4^{q}	1.6 ± 0.3^{p}	2.7 ± 0.2^{q}	<0.001	0.361	0.841

hsp90aa	1.0 ± 0.1 [c]	1.4 ± 0.3 [bc]	3.3 ± 0.6 [a]	1.0 ± 0.2 [c]	1.5 ± 0.5 [bc]	3.8 ± 1.3 [a]	1.8 ± 0.4 [abc]	3.0 ± 0.9 [ab]	0.508	0.128	0.007
hsp90b	1.0 ± 0.1 [p]	1.2 ± 0.1 [q]	1.0 ± 0.1 [p]	1.6 ± 0.3 [q]	0.8 ± 0.0 [p]	1.1 ± 0.1 [q]	1.0 ± 0.1 [p]	1.2 ± 0.1 [q]	0.002	0.106	0.617

Different subscription letters indicate statistical differences in stress (p, q), treatment (x, y, z) and interaction of treatment and stress (a, b, c, d, e) in the expression of antioxidant capacity biomarker.

Table S3. The response of several oxidative stress-related biomarkers of Senegalese sole fed with different diets (CTRL, CC, GT and GS) at the end of the growth trial (standard) and after thermal chronic stress (chronic).

	CTRL		CC		GT		GS		2-way ANOVA		
	Standard	Chronic	Standard	Chronic	Standard	Chronic	Standard	Chronic	Stress	Diet	Diet*Stress
Physiological indicators											
CAT (μmol/min/mg protein)	13.3 ± 0.8^{p}	15.9 ± 2.5^{q}	13.2±0.9^{p}	17.1±3.6^{q}	12.6±1.0^{p}	16.2±2.3^{q}	12.2±1.3^{p}	17.0±3.1^{q}	<0.001	0.778	0.531
GST (nmol/min/mg protein)	21.5 ± 2.5^{px}	20.1 ± 2.7^{qx}	18.1±1.6^{py}	16.9±2.1^{qy}	17.9±2.7^{py}	14.8±1.0^{qy}	16.0±3.1^{py}	16.1±0.8^{qy}	0.012	<0.001	0.246
GSH (μM/min/mg protein)	102.9 ± 11.9^{a}	15.2 ± 10.0^{e}	97.9 ± 4.7^{a}	14.5 ± 1.7^{e}	39.6 ± 6.9^{b}	24.2 ± 3.8^{de}	36.6 ± 4.4^{bc}	26.6 ± 5.6^{cd}	<0.001	<0.001	<0.001
TAC (μM Trolox equivalents/mg protein)	1.3 ± 0.2^{p}	6.0 ± 2.1^{q}	1.3 ± 0.4^{p}	5.6 ± 1.3^{q}	1.5 ± 0.4^{p}	4.5 ± 0.5^{q}	1.1 ± 0.1^{p}	3.9 ± 3.4^{q}	<0.001	0.229	0.256
HSP70 (μg/mg protein)	1.3 ± 0.1^{bc}	1.5 ± 0.4^{ab}	0.9 ± 0.3^{cd}	1.6 ± 0.5^{ab}	1.6 ± 0.4^{ab}	1.5 ± 0.2^{ab}	0.6 ± 0.1^{d}	1.9 ± 0.4^{a}	<0.001	0.097	<0.001
LPO (nmol TBARS/mg protein)	6.7 ± 1.0^{py}	1.8 ± 0.4^{qy}	6.2 ± 0.9^{py}	1.9 ± 0.5^{qy}	8.0 ± 1.7^{px}	2.9 ± 0.8^{qx}	6.6 ± 0.7^{pxy}	3.1 ± 0.3^{qxy}	<0.001	<0.001	0.090
PC (nmol carbonyl/mg protein)	29.5 ± 11.4^{d}	35.8 ± 16.5^{cd}	23.0 ± 10.1^{d}	37.2 ± 12.5^{cd}	66.7 ± 16.0^{b}	25.6 ± 10.3^{d}	58.7 ± 7.5^{bc}	107.1 ± 37.3^{a}	0.114	<0.001	<0.001
Gene expression											
sod3	1.0±0.1	0.9±0.1	0.9±0.1	1.2±0.1	0.8±0.1	1.2±0.2	1.0 ± 0.1	1.1 ± 0.1	0.053	0.830	0.270
cat	1.0±0.1^{pxy}	1.6±0.2^{qxy}	1.1±0.1^{pxy}	2.0±0.2^{qxy}	0.9±0.1^{py}	1.5 ± 0.1^{qy}	1.4 ± 0.1^{px}	1.8 ± 0.2^{qx}	<0.001	0.021	0.310
gsr	1.0 ± 0.0^{p}	0.5 ± 0.0^{q}	1.2 ± 0.2^{p}	0.5 ± 0.1^{q}	0.9 ± 0.2^{p}	0.4 ± 0.0^{q}	1.0 ± 0.2^{p}	0.5 ± 0.0^{q}	<0.001	0.078	0.644
gpx1	1.0 ± 0.1^{c}	2.0 ± 0.1^{a}	1.9 ± 0.5^{ab}	1.8 ± 0.1^{ab}	1.1 ± 0.1^{bc}	2.6 ± 0.3^{a}	1.6 ± 0.2^{abc}	2.0 ± 0.2^{a}	<0.001	0.161	0.007
gpx3	1.0 ± 0.1^{p}	1.9 ± 0.3^{q}	0.9 ± 0.1^{p}	1.8 ± 0.4^{q}	0.9 ± 0.1^{p}	2.1 ± 0.3^{q}	1.1 ± 0.2^{p}	1.6 ± 0.1^{q}	<0.001	0.854	0.335
hsp70	1.0 ± 0.1^{p}	1.8 ± 0.2^{q}	1.2 ± 0.2^{p}	2.5 ± 0.3^{q}	1.3 ± 0.1^{p}	1.4 ± 0.3^{q}	1.6 ± 0.3^{p}	2.8 ± 1.1^{q}	0.002	0.112	0.251
hsp90aa	1.0 ± 0.1^{ab}	1.6 ± 0.4^{ab}	3.3 ± 0.6^{a}	0.7 ± 0.1^{b}	1.5 ± 0.5^{ab}	0.9 ± 0.2^{b}	1.8 ± 0.4^{ab}	1.3 ± 0.4^{ab}	0.015	0.483	0.020

hsp90b	1.0 ± 0.1^{p}	0.8 ± 0.1^{q}	1.0 ± 0.1^{p}	0.6 ± 0.1^{q}	0.8 ± 0.0^{p}	0.8 ± 0.1^{q}	1.0 ± 0.1^{p}	0.9 ± 0.1^{q}	0.005	0.346	0.511

Different subscription letters indicate statistical differences in stress (p, q), treatment (x, y, z) and interaction of treatment and stress (a, b, c, d, e) in the expression of antioxidant capacity biomarker.
